# Comparison of contemporaneous Sentinel-2 and EnMAP data for vegetation index-based estimation of leaf area index and canopy closure of a boreal forest

**DOI:** 10.1080/22797254.2024.2432975

**Published:** 2024-11-27

**Authors:** Jussi Juola, Aarne Hovi, Miina Rautiainen

**Affiliations:** Department of Built Environment, School of Engineering, Aalto University, Aalto, Finland

**Keywords:** Coniferous, LAI, canopy cover, EnMAP, Sentinel-2, hyperspectral

## Abstract

Data from the new hyperspectral satellite missions such as EnMAP are anticipated to refine leaf area index (LAI) or canopy closure (CC) monitoring in conifer-dominated forest areas. We compared contemporaneous multispectral and hyperspectral satellite images from Sentinel-2 MSI (S2) and EnMAP and assessed whether hyperspectral images offer added value in estimating LAI, effective LAI (LAIeff), and CC in a European boreal forest area. The estimations were performed using univariate and multivariate generalized additive models. The models utilized field measurements of LAI and CC from 38 forest plots and an extensive set of vegetation indices (VIs) derived from the satellite data. The best univariate models for each of the three response variables had small differences between the two sensors, but in general, EnMAP had more well-performing VIs which was reflected in the better multivariate model performances. The best performing multivariate models with the EnMAP data had ~1–6% lower relative RMSEs than S2. Wavelengths near the green, red-edge, and shortwave infrared regions were frequently utilized in estimating LAI, LAIeff, and CC with EnMAP data. Because EnMAP could estimate LAI better, the results suggest that EnMAP may be more useful than multispectral satellite sensors, such as S2, in monitoring biophysical variables of coniferous-dominated forests.

## Introduction

Canopy closure (CC) and leaf area index (LAI) are essential metrics in characterizing the forest canopy: they play a pivotal role in the biological and physical processes of a forest ecosystem, influencing everything from radiation to microclimate dynamics (Chen et al., 1997; Jennings et al., 1999). CC is defined as the fraction of the sky hemisphere obscured by vegetation when observed from a single point (Jennings et al., 1999) and is a key parameter in determining whether an area qualifies as a forest according to the Food and Agriculture Organization of United Nations’ definition (Forest Resources Assessment, 2015). LAI, in turn, is defined as the hemisurface area of green leaves and needles per unit ground surface area [m^2^m^−2^] (Chen & Black, 1992; Lang, 1991), and is one of the essential climate variables (Global Climate Observing System GCOS, 2024) needed as input for land cover monitoring and forest ecosystem modelling.

Remote sensing allows monitoring CC and LAI at local, regional, and global scales. Operational monitoring of LAI at a global or continental scale has been conducted for over two decades (e.g. Myneni et al., 2002; Yan et al., 2018; Yang et al., 2017), and substantial international efforts have been targeted at validation of satellite-based LAI products so that their quality across vegetation biomes would be understood (e.g. Abuelgasim et al., 2006; Chen et al., 2002; Li et al., 2022; Morisette et al., 2006). While the global LAI products form a valuable basis for analyzing global trends in vegetation (e.g. Pu et al., 2024; Zhu et al., 2016), they have a coarse spatial resolution which does not always match the small-scale changes occurring in the forest due to, for example, forest management and disturbances. Maps of CC, on the other hand, have also been produced for over two decades under different names such as vegetation continuous fields or tree cover (e.g. Hansen et al., 2002; Sexton et al., 2013), and despite varying definitions (Majasalmi & Rautiainen, 2021), are utilized in many land surface model components of climate models. While some of these CC-related products have relatively high spatial resolution (e.g. Hansen et al., 2013), they are not generated at the same high temporal resolution (daily or weekly) as the LAI products.

Applications needing frequently produced local and regional maps of LAI or CC at high spatial resolution call for developing methods based on Landsat and Sentinel-2 data (e.g. Chen & Cihlar, 1996; Hadi et al., 2016; Korhonen et al., 2017; Majasalmi & Rautiainen, 2016). An example of such high spatial resolution needs comes from managed European boreal forests where the typical size of a forest compartment ranges from only 1 to 2 ha. An open question is if high spectral resolution data, i.e. hyperspectral (imaging spectroscopy) satellite data provided by the new and forthcoming missions could refine high spatial resolution LAI or CC monitoring, and furthermore, whether it would provide additional value in conifer-dominated areas where especially LAI monitoring is challenging due to clumped canopy structures (e.g. Chen et al., 1997; Stenberg et al., 2008). The latest generation of Earth Observation missions, such as EnMAP (Environmental Mapping and Analysis Program; Storch et al., 2023), now supply hyperspectral data to the scientific community – the first EnMAP images became available during 2023. In comparison to, for example, Sentinel-2, which captures imagery with high spatial resolution but limited spectral bands, EnMAP captures both high spatial and spectral resolution data.

Data from the new hyperspectral satellite missions, such as EnMAP, are anticipated to address three key challenges previously associated with using broadband vegetation indices (VI) in estimating biophysical variables: saturation at LAI values higher than 3–4 (Myneni et al., 1997), susceptibility to variation in tree species composition (Chen et al., 2002), and sensitivity to understory vegetation (Eriksson et al., 2006; Nemani et al., 1993). Previous research has already shown that the two broad shortwave infrared (SWIR) bands from the multispectral Landsat 7 ETM+ sensor reduced these three effects when used in a VI (Stenberg et al., 2004). Only few studies have explored the potential added value of hyperspectral data in estimating biophysical variables of conifer-dominated forest canopies. For boreal forests, Halme et al. (2019) showed that the additional value of using airborne hyperspectral remote sensing data in monitoring biophysical variables is mainly associated with variables which contain species-specific information, e.g. LAI. The use of earlier available satellite-borne hyperspectral data from EO-1 hyperion has been investigated in conifer-dominated forests by Gong et al. (2003) and Heiskanen et al. (2013), who found that narrowband VIs, particularly those utilizing wavelengths in the SWIR and some in the near-infrared (NIR) regions, were more effective for LAI estimation than conventional broadband VIs utilizing red and NIR bands. Heiskanen et al. (2013) also suggested that the improved LAI estimates with narrowband VIs for boreal forests were primarily due to finer-scale spectral sampling in the NIR wavelengths that have water absorption features. The challenge with hyperspectral data is to identify the most accurate spectral transformation sensitive to biophysical variables from the hundreds of contiguous spectral bands. Despite the above-mentioned studies, it is currently unclear which narrowband VIs or their combinations are most sensitive to boreal forest CC and LAI, and to what extent can narrowband VIs outperform broadband VIs, if at all. A common challenge when comparing the utility of multi- and hyperspectral satellite data arises from the temporal discrepancy between images: a multispectral and hyperspectral satellite image is rarely available from the same time. This challenge is prominent especially in the high latitudes, where the short growing season and fast phenological changes make it important to perform the comparison with contemporaneous hyperspectral and multispectral satellite data.

In this research paper, we compare contemporaneous multispectral and hyperspectral satellite data for estimating CC and LAI of a boreal forest area. The study is based on field measurements of LAI and CC, and Sentinel-2 MSI and EnMAP images which were acquired approximately 13 minutes apart. We assessed whether the EnMAP data offers added value compared to Sentinel-2 MSI in estimating LAI and CC. To our knowledge, this is the first study evaluating the potential of EnMAP in monitoring boreal forest LAI and CC.

## Materials and methods

### Study area and field measurements

Our coniferous-dominated study area was in Hyytiälä, Finland (61°51′N, 24°18′E) with moderately flat terrain (130–200 m a.s.l.). The forests are mainly managed with common forestry practices, except for a few protected forests. The field data comprise 38 forest plots that were selected to cover maximal variation in LAI, CC, and tree species composition. The structure of forests in this area ranged from medium sparse to dense. The plots were on both mineral (*n* = 31) and organic (*n* = 7) soils.

Measurements in the plots were conducted between 18 July and 1 August in 2023. We measured forest characteristics using relascope sampling. These measurements were used for providing descriptive information on the forest characteristics (Table A1 in Appendix A) and to obtain tree species proportions in the 38 plots. There were 27 conifer-dominated (conifer share >80%), 5 broadleaved-dominated, and 6 mixed species plots. Reference measurements of LAI and CC were obtained from hemispherical photographs taken near dusk or dawn, or during overcast sky conditions. We took 12 photographs per plot, using a cross-shaped sampling scheme, in which three photographs were taken in each cardinal compass direction, at 2 m, 6 m, and 10 m from the plot center (Majasalmi et al., 2012). The height of the camera from ground was 1 m. We used a Nikon D5600 camera equipped with a geometrically calibrated Sigma 4.5 mm f/2.8 DC HSM Circular Fisheye Lens. The photographs were taken in jpeg format with the highest possible quality. The blue band of the photographs was binarized using an algorithm of Nobis and Hunziker (2005). The binarized images were then divided into five concentric rings between zenith angles of 0° and 75°. Mean canopy gap fraction at each study plot and zenith ring was calculated as the sum of canopy pixels divided by the total number of pixels. CC was calculated as one minus canopy gap fraction at the highest zenith ring (0°–15°). Effective LAI (LAIeff) was calculated using gap fractions of all zenith rings, with the same principle as employed in the LAI-2200 instrument (LI-COR, 2012). Clumping-corrected LAI (hereafter LAI) was obtained by dividing LAIeff with a shoot clumping coefficient: 0.58 for pine (Smolander et al., 1994), 0.64 for spruce (Stenberg et al., 1995), and 1 for broadleaved trees. In mixed species plots, species-weighted average clumping coefficient was used.

### Sentinel-2 MSI and EnMAP satellite data

We used one preprocessed image from Sentinel-2B MSI (hereafter S2) and one from EnMAP, both acquired within a 13-minute time interval on 20 August 2023, at 12:53:30 and 13:06:42 local time, respectively (Table 1). The S2 image is a Level 2A product (processing baseline 05.09) provided by the European Space Agency. The acquisition and processing of the EnMAP image were ordered through the EnMAP instrument planning portal (EnMAP, 2024). The preprocessing parameters used in our order are reported in Appendix B

**Table 1.** Image details for the sentinel-2 and EnMAP data.

	Sentinel-2B MSI	EnMAP
Image ID	S2B_MSIL2A_20230820T094549_N0509_R07	ENMAP01-L2A-
	9_T35VLJ_20230820T124817	DT0000038765_20230820T100640Z_001_V010402_20
		20240903T210937Z
Sun zenith angle^1^	49.7°	49.5°
View zenith angle^1^	5°	18°
Processing level	L2A (orthorectified and atmospherically corrected data)	L2A (orthorectified and atmospherically corrected data)
Reflectance quantity	Hemispherical-directional reflectance factor (HDRF)	Hemispherical-directional reflectance factor (HDRF)
Reference system	WGS84 / UTM Zone 35	WGS84/ UTM Zone 35
Provider	European Space Agency	German Space Agency
Other details	Processing baseline 05.09	EnMAP data ©DLR 2023

For S2, we utilized all 12 bands available from the L2A product, with center wavelengths at 442, 492, 559, 665, 704, 739, 780, 833, 864, 943, 1610, and 2186 nm. We used bands at their native spatial resolution, and thus the pixel size was 10, 20, or 60 m depending on the band. For EnMAP, the pixel size was 30 m, and there were 219 bands that covered wavelengths 418–1331, 1449–1780, and 1968–2445 nm. The average spectral sampling distance was 6.4 nm below 993 nm and 10 nm elsewhere. We calculated the mean hemispherical-directional reflectance factor (HDRF, Schaepman-Strub et al., 2006) of S2 and EnMAP pixels that had centers overlapping with a 60 m × 60 m rectangle around the plot center. A 60 m × 60 m area was selected to have the same surface area for analysis for both sensors, irrespective of the band’s native spatial resolution. Hence, for S2, we used the mean HDRF of 36, 9, or 1 pixels, depending on the pixel size. For EnMAP, we used the mean HDRF of 4 pixels per band. The images were visually examined against each other, and against high-resolution aerial orthoimages, revealing sub-pixel geometric accuracy. Scene classification layers in both images were examined and indicated no clouds and cloud shadows or saturated or defective pixels.

### Estimation of leaf area index and canopy closure from satellite data

#### Predictor variables

As predictors for LAI, LAIeff and CC, we used HDRFs at individual spectral bands from both S2 and EnMAP, along with an extensive set of different VIs (Table C1 in Appendix C). For S2, we tested all possible band combinations of several widely used VI forms, including normalized difference index (NDI), simple ratio (SR), derivative (D), Reduced Simple Ratio (RSR) with all possible SR permutations, and various three-band indices (modified normalized difference index (mND), modified simple ratio (mSR), Tian’s three-band spectral index (3BSIT), Verrelst’s three-band spectral index (3BSIV), Wang’s three-band spectral index (3BSIW)) (see Appendix C for formulas). For EnMAP, we computed all possible band combinations of NDI, SR, D, RSR, and a selection of commonly used narrowband VIs (CIgreen, CIrededge, MCARI, MCARI1, MCARI2, CAI, and PSRI) (see Appendix C for formulas). We omitted the different three-band indices for EnMAP due to the number of permutations making them computationally infeasible. When computing the RSR, we calculated the minimum and maximum SWIR HDRFs from data recorded for our field plots (Brown et al., 2000) using 1610 nm for S2 and 1609 nm for EnMAP (0.056 and 0.194, and 0.064 and 0.205, respectively). The total number of predictor variables available for selection for S2 and EnMAP were 7140 and 191,194, respectively.

#### Models for LAI, LAIeff, and CC

In remote sensing applications, GAMs have been used to predict CC or tree cover (e.g. Halperin et al., 2016; Staver et al., 2011) and estimate LAI (e.g. Korhonen et al., 2017). We used GAMs to model the response variables LAI, LAIeff, and CC with a sum of smoothing functions (Wood, 2017)

(1) $$\begin{array}{c} y=\beta_{0}+\overset{p}{\underset{j=1}{\sum }}f_{j}\left(X_{j}\right)+\varepsilon \end{array}$$

where

y is the response variable,

*β*_0_ is the model intercept,

*f*_*j*_(*X*_*j*_) is a smooth function of predictor *X*_*j*_,

and *ε* is the residual.

For LAI and LAIeff, we used Gamma GAMs with inverse link function to ensure that our model predictions were non-negative. For CC, we utilized quasibinomial GAMs with a logistic link function to have predictions within a unit interval.

First, we fitted univariate models for LAI, LAIeff, and CC, testing all predictor variables. We used root mean square error (RMSE), relative RMSE (RMSE-%), and adjusted coefficient of determination (Adj. *R*^2^) to evaluate the performance of the models. Next, we fitted multivariate GAM models for combinations of two and three predictors. The maximum number of predictors was restricted to three due to computational complexity, given the large number of predictor variables. Korhonen et al. (2017) observed that RMSEs stabilized around 3–4 predictors with S2 data. The selection of predictor variables was performed with simulated annealing (SA) (Kirkpatrick et al., 1983), which has been demonstrated as the most accurate method for selecting predictors in remote sensing-based forest inventory (Packalén et al., 2012). SA is a randomized search method used to approximate the global optimum within a large search space, aiming for a near-optimal solution. SA allows for probabilistic transitions to worse solutions within the search space to avoid local optima. The likelihood of accepting worse solutions is controlled by the parameter known as temperature, which gradually decreases according to a cooling schedule.

In this study, neighbor solutions or random transitions for both multivariate schemes were generated by randomly changing one predictor variable at a time. Initial temperatures (*T*_*0*_) for each response variable were computed with $T_{0}=-\frac{\overset{ˉ}{\mathrm{\Delta E}}}{\ln\left(X_{0}\right)}$ (Johnson et al., 1989). We set *X*_*0*_ (initial acceptance ratio) to 0.8 and computed $\overset{ˉ}{\Delta E}$ (average cost) for a set of 500 random transitions. We used a geometric cooling schedule with a rate of 0.96 and used random starting solutions to initialize the simulations. The cost function minimized with SA was the RMSE. We ran 500 outer-loop iterations (temperature decrements) and 500 inner-loop iterations (transitions at each temperature), resulting in a total of 250,000 steps for each run. Given the stochastic nature of SA, we repeated the variable selection 50 times for each of the two and three predictor models per sensor.

All the modeling was implemented with R Statistical Software (v4.3.2; R Core Team, 2024) and the R package mgcv version 1.9–1.

## Results and discussion

### Differences between S2 and EnMAP bottom-of-atmosphere reflectances

**Figure 1.**
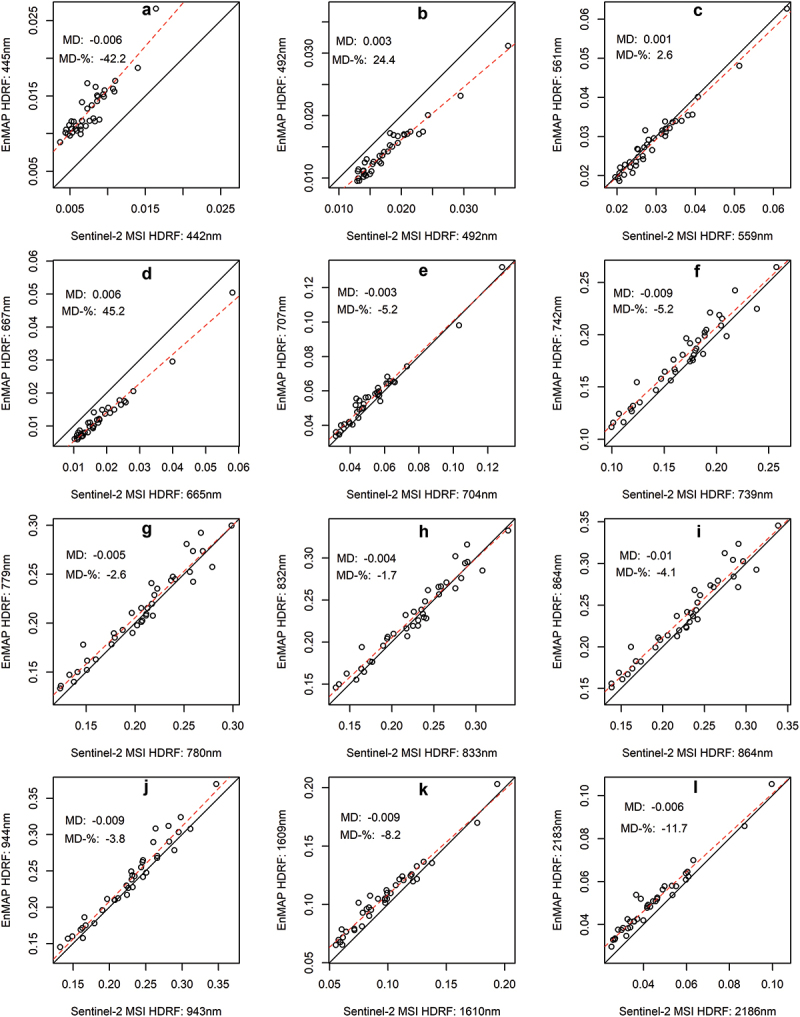
A comparison of broadband sentinel-2 bottom-of-atmosphere hemispherical-directional reflectance factors (HDRF) to the closest EnMAP narrowband HDRFs in field plots (black circles). Each subfigure visualizes the one-to-one line (black solid line) and a linear regression between EnMAP and sentinel-2 hDRFs (red dashed line). MD is mean difference (sentinel-2 minus EnMAP), and MD-% is relative MD.

In general, S2 had a lower bottom-of-atmosphere HDRF than EnMAP when comparing the 12 closest center wavelengths for the 38 field plots (Figure 1). The majority of the 12 bands had relative mean differences (MD-%) ranging from −1.7% to −42.2% (Figure 1). However, S2 had higher HDRF than EnMAP for the blue (Figure 1b), green (Figure 1c) and red (Figure 1d) wavelengths. The two largest MD% between S2 and EnMAP HDRFs were at ultra blue (Figure 1a) and red (Figure 1d) wavelengths. The NIR wavelength had a very close to one-to-one relationship between S2 and EnMAP HDRFs (Figure 1h). The differences between EnMAP and S2 were mainly systematic (Figure 1). The results indicate that S2 and EnMAP are strongly correlated, and it is likely that the systematic errors observed between the two sensors were caused by the differences in spectral band width, view zenith angle, and uncertainties and differences in atmospheric and topographic correction implemented by the data providers.

### S2 and EnMAP univariate estimations of LAI, LAIeff, and CC

**Figure 2.**
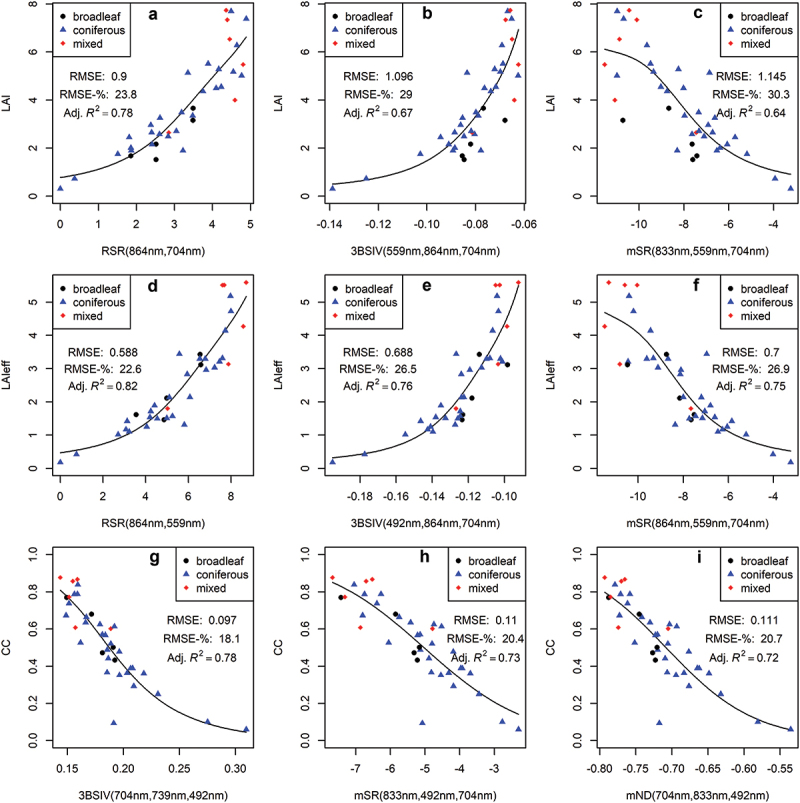
The top three vegetation index-based predictors for sentinel-2 data, and their univariate generalized additive model (GAM) fits for three response variables: leaf area index (LAI, A – C), effective LAI (LAIeff, D – F), and canopy closure (CC, G – I). The x-axis labels indicate the vegetation index and the wavelengths it is based on: reduced simple ratio (RSR), Verrelst’s three-band spectral index (3BSIV), modified simple ratio (mSR), and modified normalized difference index (mND). Each subplot (A – I) shows the root mean squared error (RMSE), relative RMSE (RMSE-%), and adjusted coefficient of determination (adj. *R*^2^) for the model fit.

**Figure 3.**
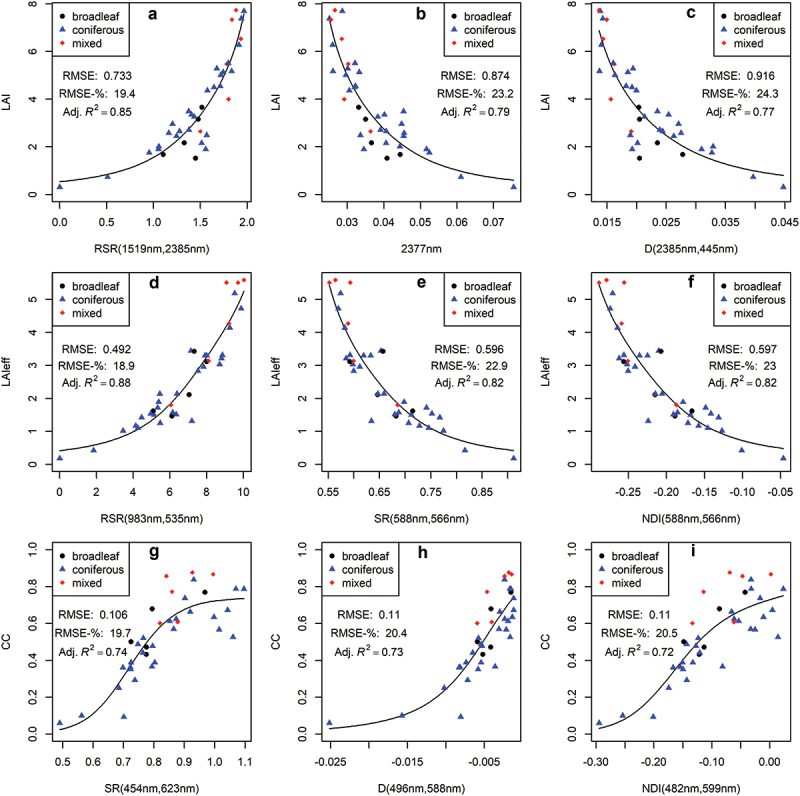
The top three vegetation index-based predictors for EnMAP data, and their univariate generalized additive model (GAM) fits for three response variables: leaf area index (LAI, A – C), effective LAI (LAIeff, D – F), and canopy closure (CC, G – I). The x-axis labels indicate the vegetation index and the wavelengths it is based on: reduced simple ratio (RSR), simple ratio (SR), normalized difference index (NDI), and derivative (D). Each subplot (A – I) shows the root mean squared error (RMSE), relative RMSE (RMSE-%), and adjusted coefficient of determination (adj. *R*^2^) for the model fit.

The best-performing VIs derived from S2 and EnMAP data, when used in univariate models, had strong correlations with all three response variables (Figures 2 and 3). When estimating LAI and LAIeff, the narrowband EnMAP VIs had lower relative RMSEs (~19–24%) compared to the broadband S2 VIs (~23–30%) (Figures 2a–f and 3a–f). The optimal RSR, utilizing SWIR reflectance near 1610 nm, had the smallest RMSE-% for both S2 and EnMAP when modelling LAI and LAIeff (Figures 2a,d and 3a,d respectively). The other top two performing VIs for LAI and LAIeff with S2 were combinations of three broadbands (3BSIV and mSR), utilizing wavelengths from the blue, green, red-edge, and NIR regions (Figure 2a–f). For EnMAP, a single wavelength in the SWIR region and the D index, featuring wavelengths near the blue and SWIR regions, were second and third best for LAI estimations (Figure 3a–c). Similarly, the SR and NDI indices, featuring wavelengths near the green region, were the second and third best for LAIeff estimations (Figure 3d–f). The modeling results for LAI and LAIeff with best-performing predictors from each predictor category per S2 and EnMAP are provided in Tables D1 and D2 in Appendix D.

S2 performed better than EnMAP in estimating CC with the best univariate GAM. The top three VIs for both S2 and EnMAP had RMSE-%s ranging from 18.1% to 20.7% and from 19.7% to 20.5%, respectively (Figures 2G–I and 3G–I). For S2, the top three VIs (3BSIV, mSR, and mND) for CC estimation were three-band combinations utilizing wavelengths from the blue, red-edge, and NIR regions (Figure 2g–i). Meanwhile, EnMAP’s top three VIs (SR, D, and NDI) for CC estimation featured wavelengths between the blue, green, and red wavelength regions (Figure 3g–i). The better performance of S2 in univariate estimation of CC could be associated with having a view zenith angle closer to nadir than EnMAP (Table 1). The modeling results for CC with all predictor categories per S2 and EnMAP can be found in Table D3 in Appendix D.

The top-performing VIs for S2 had moderately better univariate estimations for LAIeff than LAI (Figure 2), whereas EnMAP VIs performed similarly or equally for both LAI and LAIeff (Figure 3). Hence, EnMAP could be better than S2 in estimating LAI over coniferous-dominated boreal forests that typically have clumped canopy structures. These results correspond with the findings of Halme et al. (2019) who concluded, based on airborne imaging spectroscopy, that the added value of hyperspectral data is associated with biophysical variables that contain species-specific information, such as LAI.

### S2 and EnMAP multivariate estimations of LAI, LAIeff, and CC

Multivariate GAMs showed clearly better prediction accuracies compared to the univariate GAMs (Tables D1–3 in Appendix D, Table 2, and Table E1 in Appendix E). In general, the multivariate GAMs with two and three predictors from EnMAP data, selected using SA, had lower median RMSE-% (~9–13%) compared to S2 (~11–19%) when estimating LAI, LAIeff, and CC (Table 2). S2 had better performance than EnMAP when estimating CC with two predictors, though the difference was small (~0.4% in absolute values), and EnMAP did find a lower minimum RMSE than S2 during the variable selection (Table 2). For the three-predictor models, EnMAP produced lower median RMSE-%s and had smaller minimum and maximum RMSEs across 50 runs per predictor and response variable compared to S2 (Table 2). Even though the search space with EnMAP was extensive compared to S2, the SA algorithm was able to find better predictor combinations, highlighting the higher number of well-performing two-band VIs from hyperspectral EnMAP data. This follows the same trend of results as seen with the univariate estimations. LAIeff (~10–19%) was the most difficult biophysical variable to predict out of the three, for both sensors.

**Table 2.** The median, relative median, standard deviation (sd), minimum (min), and maximum (max) root mean squared errors (RMSEs) and adjusted coefficients of determination (adj. *R*^2^) from 50 runs of simulated annealing variable selection per response variable and number of predictors derived from sentinel-2 and EnMAP data.

		Sentinel-2 MSI					EnMAP				
		RMSE					RMSE				
Response variable	Number of predictors	median	median [%]	sd	min	max	median	median [%]	sd	min	max
LAI	2	0.64	16.88	0.032	0.60	0.71	0.46	12.23	0.063	0.36	0.54
	3	0.57	14.98	0.042	0.42	0.63	0.35	9.17	0.052	0.26	0.45
LAIeff	2	0.49	18.85	0.010	0.48	0.51	0.33	12.81	0.033	0.26	0.38
	3	0.41	15.88	0.044	0.29	0.46	0.27	10.28	0.034	0.19	0.31
CC	2	0.07	12.94	0.001	0.07	0.07	0.07	13.36	0.004	0.06	0.08
	3	0.06	11.44	0.003	0.05	0.07	0.05	9.95	0.008	0.04	0.07
		Adj. *R*^2^					Adj. *R*^2^				
LAI	2	0.88		0.012	0.85	0.9	0.94		0.016	0.91	0.96
	3	0.9		0.013	0.88	0.95	0.96		0.011	0.94	0.98
LAIeff	2	0.87		0.005	0.86	0.88	0.94		0.011	0.92	0.96
	3	0.9		0.019	0.88	0.95	0.96		0.010	0.94	0.98
CC	2	0.88		0.004	0.87	0.89	0.87		0.014	0.86	0.91
	3	0.9		0.009	0.89	0.93	0.93		0.021	0.89	0.96

**Figure 4.**
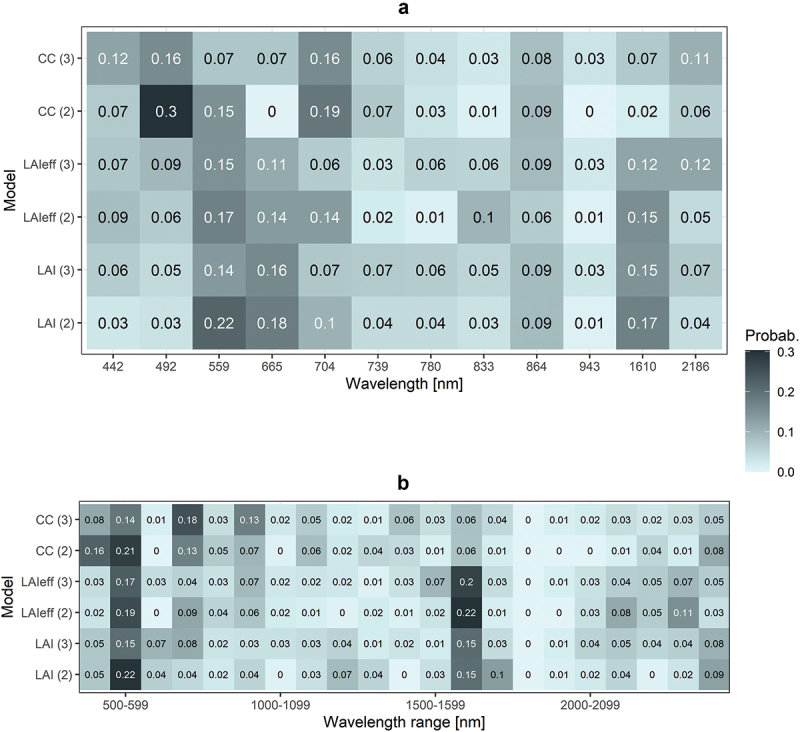
The wavelengths for sentinel-2 (A) and wavelength ranges for EnMAP (B) that were most frequently selected across 50 runs of simulated annealing variable selection when estimating LAI, LAIeff, and CC using two (2) and three (3) wavelengths or vegetation indices as predictors.

In general, for S2, the most frequently featured wavelengths in the 50 solutions of SA variable selection were similar for LAI and LAIeff but differed slightly for CC estimations (Figure 4a). For LAI and LAIeff, the multivariate models favored the green, red, NIR and SWIR wavelengths (Figure 4a). This is not surprising as these wavelengths are highly correlated with the amount of green vegetation. In contrast, for CC, the blue bands and the beginning of the red-edge region were selected often, which can be attributed to the high absorption of foliage pigments (Figure 4a). Similarly, for EnMAP, wavelengths were often selected from near the green, red-edge, and mid-SWIR regions for all three variables (Figure 4b). Also, wavelengths from 2100–2400 nm were relatively often selected for LAI and LAIeff estimations with EnMAP VIs (Figure 4b). In contrast, for CC estimations with EnMAP, wavelengths were frequently selected from the blue and NIR regions (Figure 4b) which could be associated with moisture or pigment absorption features detectable by finer-scale spectral resolution, as suggested by Heiskanen et al. (2013). The high frequencies at 1610 nm (S2) and 1600–1700 nm (EnMAP) wavelength regions (Figure 4) are partly caused by the RSR VI that performed very well for both sensors. Future studies should focus on developing and testing VIs tailored for estimating canopy biophysical variables, utilizing data from the new and upcoming hyperspectral satellite sensors. These indices should incorporate multiple narrow spectral bands to optimize their effectiveness while also considering the high-computing resources necessary for their application.

## Conclusions

We compared contemporaneous Sentinel-2 MSI (S2) and EnMAP satellite images and assessed whether hyperspectral images offer added value in estimating leaf area index (LAI), effective LAI (LAIeff), and canopy closure (CC) in a European boreal forest area. The estimations were performed using univariate and multivariate generalized additive models (GAMs). The regression models utilized field measurements of LAI and CC from 38 forest plots and an extensive set of vegetation indices (VIs) derived from the satellite data. The best univariate models for each of the three response variables had small differences between the two sensors, but in general, EnMAP had more well-performing VIs which was reflected in the better multivariate model performances. The best performing multivariate models utilizing hyperspectral EnMAP data had ~1–6% lower relative RMSEs than S2 when estimating LAI, LAIeff, and CC. Wavelengths near the green, red-edge, and shortwave infrared regions were frequently utilized in estimating LAI, LAIeff, and CC with EnMAP data. Because EnMAP could estimate LAI better, the results suggest that the finer spectral resolution from EnMAP’s imaging spectroscopy may be more useful than multispectral satellite sensors, such as S2, in monitoring biophysical variables of coniferous-dominated forests.

## Data Availability

The EnMAP and Sentinel-2 satellite data used in this study are available from the EnMAP instrument planning portal (https://planning.enmap.org/) and the Copernicus Browser (https://browser.dataspace.copernicus.eu/), respectively. The field data are available from the corresponding author, JJ, upon reasonable request.

## Appendix A

**Table A1.** Forest characteristics in the 38 study plots.

Characteristic	Mean (min – max)
Basal area [m^2^ m^−2^], of which	23 (3–51)
dead trees [%]	4 (0–23)
Basal area of living trees [m^2^ m^−2^], of which	22 (3–46)
pine [%]	42 (0–100)
spruce [%]	38 (0–100)
birch [%]	19 (0–96)
other broadleaved trees [%]	0 (0–5)
Diameter at breast height [cm]	21 (5–48)
Tree height [m]	19 (6–37)
Effective leaf area index [m^2^ m^−2^]	2.6 (0.2–5.6)
Leaf area index [m^2^ m^−2^]	3.8 (0.3–7.7)
Canopy closure [0–1]	0.54 (0.06–0.88)

## Appendix B. Parameters applied when ordering the EnMAP image

Acquisition and processing of the EnMAP image were ordered through the EnMAP instrument planning portal (EnMAP, 2024). We ordered the image in GeoTIFF format and selected the atmospheric correction for land surfaces (de Los Reyes et al., 2023). The resampling method applied in the orthorectification was bilinear interpolation. We selected no cirrus or haze removal, no interpolation of missing bands, and automatic terrain (topographic) correction. The season, which affects the atmospheric profile used in the atmospheric correction, was selected as summer, and the retrieval of atmospheric ozone column was automatic. Sunglint correction parameters were according to the scenario “Sunglint Avoidance not to be considered”, explained in the instrument planning portal user guide (Pinnel & Carmona, 2022). This was because we studied the land surface where sunglint is not a problem.

## Appendix C. Vegetation index formulas

**Table C1.** A list of vegetation indices and their respective formulas that were used in the modelling.

Number	Name	Abbr.	Formula	Reference (DOI)
1	Derivative	D	B1-B2	10.2307/1936256
2	Simple Ratio	SR	B1/B2	10.1016/j.rse.2003.09.004
3	Normalized Difference Index	NDI	(B1-B2)/(B1+B2)	10.1016/j.rse.2003.09.004
4	Reduced Simple Ratio	RSR	(B1/B2) × [(ρ_SWIR_max_-ρ_SWIR_)/(ρ_SWIR_max_-ρ_SWIR_min_)]	10.1016/S0034-4257(99)00035-8
5	Modified Normalized Difference Index	mND	(B1-B2)/(B1+B2-2×B3)	10.1016/j.rse.2003.09.004
6	Modified Simple Ratio	mSR	(B1-B3)/(B2-B3)	10.1016/j.rse.2003.09.004
7	Tian’s Three-Band Spectral Index	3BSIT	(B1-B2-B3)/(B1+B2+B3)	10.1007/s11104-013-1937-0
8	Verrelts’s Three-Band Spectral Index	3BSIV	(B1-B3)/(B2+B3)	10.1016/j.isprsjprs.2015.04.013
9	Wang’s Three-Band Spectral Index	3BSIW	(B1-B2 + 2×B3)/(B1+B2-2×B3)	10.1016/j.fcr.2012.01.014
10	CIgreen		(ρ_773–803_/ρ_518–579_)-1	10.1029/2002GL016450
11	CIrededge		(ρ_773–803_/ρ_702–742_)-1	10.1029/2002GL016450
12	MCARI		[(ρ700-ρ670)-0.2(ρ700-ρ550)] × (ρ700/ρ670)	10.1016/S0034-4257(00)00113-9
13	MCARI1		1.2[2.5(ρ800-ρ670)-1.3(ρ800-ρ550)]	10.1016/j.rse.2003.12.013
14	MCARI2		$\frac{1.5\left[2.5\left(\rho 800-\rho 670\right)-1.3\left(\rho 800-\rho 550\right)\right]}{\sqrt{{\left(2\times \rho 800+1\right)}^{2}-\left(6\times \rho 800-5\sqrt{\rho 670}\right)-0.5}}$	10.1016/j.rse.2003.12.013
15	CAI		0.5(ρ2021+ρ2213)-ρ2100	10.2134/agronj2001.931125x
16	PSRI		(ρ678-ρ500)/ρ750	10.1034/j.1399-3054.1999.106119.x

## Appendix D. Full results for LAI, LAIeff, and CC estimations for the different VI-based predictor categories

**Table D1.** Univariate LAI estimation results for sentinel-2 and EnMAP.

Response	Sensor	Predictor category	Wavelength(s)	RMSE	RMSE%	Adj. *R*^2^
LAI	Sentinel-2	Individual wavelength	2186 nm	1.15	30.48	0.64
	EnMAP		2377 nm	0.87	23.15	0.79
	Sentinel-2	Derivative	442 nm, 2186 nm	1.21	32.08	0.60
	EnMAP		2385 nm, 445 nm	0.92	24.26	0.77
	Sentinel-2	Simple Ratio	492 nm, 665 nm	1.26	33.4	0.56
	EnMAP		566 nm, 588 nm	0.96	25.35	0.75
	Sentinel-2	Reduced Simple Ratio	864 nm, 704 nm, 1610 nm	0.90	23.84	0.78
	EnMAP		1519 nm, 2385 nm, 1609 nm	0.73	19.41	0.85
	Sentinel-2	NDI	665 nm, 492 nm	1.30	34.45	0.54
	EnMAP		588 nm, 566 nm	0.96	25.34	0.75
	EnMAP	Individual index	CIgreen	1.65	43.60	0.26
		Individual index	CIrededge	1.46	38.65	0.42
		Individual index	MCARI	1.25	33.22	0.57
		Individual index	MCARI1	1.84	48.81	0.08
		Individual index	MCARI2	1.88	49.72	0.04
		Individual index	CAI	1.61	42.55	0.30
		Individual index	PSRI	1.50	39.86	0.38
	Sentinel-2	mSR	833 nm, 559 nm, 704 nm	1.15	30.34	0.64
		mND	833 nm, 704 nm, 559 nm	1.19	31.42	0.61
		3BSIV	559 nm, 864 nm, 704 nm	1.10	29.04	0.67
		3BSIW	780 nm, 704 nm, 492 nm	1.15	30.56	0.63
		3BSIT	492 nm, 442 nm, 665 nm	1.16	30.62	0.63

**Table D2.** Univariate LAIeff estimation results for sentinel-2 and EnMAP.

Response	Sensor	Predictor category	Wavelength(s)	RMSE	RMSE%	Adj. *R*^2^
LAIeff	Sentinel-2	Individual wavelength	442 nm	0.80	30.74	0.67
	EnMAP		2400 nm	0.62	23.73	0.80
	Sentinel-2	Derivative	442 nm, 559 nm	0.94	35.99	0.55
	EnMAP		424 nm, 2400 nm	0.64	24.66	0.79
	Sentinel-2	Simple Ratio	778 nm, 739 nm	0.83	32.00	0.65
	EnMAP		588 nm, 566 nm	0.60	22.93	0.82
	Sentinel-2	Reduced Simple Ratio	864 nm, 559 nm, 1610 nm	0.59	22.60	0.82
	EnMAP		983 nm, 535 nm, 1609 nm	0.49	18.93	0.88
	Sentinel-2	NDI	780 nm, 739 nm	0.83	32.08	0.64
	EnMAP		588 nm, 566 nm	0.60	22.97	0.82
	EnMAP	Individual index	CIgreen	1.01	38.98	0.47
		Individual index	CIrededge	0.87	33.33	0.62
		Individual index	MCARI	1.01	38.90	0.48
		Individual index	MCARI1	1.37	52.79	0.04
		Individual index	MCARI2	1.35	51.74	0.08
		Individual index	CAI	1.13	43.28	0.35
		Individual index	PSRI	1.13	43.36	0.35
	Sentinel-2	mSR	864 nm, 559 nm, 704 nm	0.70	26.91	0.75
		mND	704 nm, 864 nm, 559 nm	0.72	27.57	0.74
		3BSIV	492 nm, 864 nm, 704 nm	0.69	26.45	0.76
		3BSIW	739 nm, 1610 nm, 864 nm	0.75	29.00	0.71
		3BSIT	864 nm, 739 nm, 1610 nm	0.77	29.77	0.69

**Table D3.** Univariate CC estimation results for sentinel-2 and EnMAP.

Response	Sensor	Predictor category	Wavelength(s)	RMSE	RMSE%	Adj. *R*^2^
CC	Sentinel-2	Individual wavelength	559 nm	0.12	22.96	0.66
	EnMAP		588 nm	0.12	22.20	0.68
	Sentinel-2	Derivative	559 nm, 492 nm	0.13	23.46	0.64
	EnMAP		496 nm, 588 nm	0.11	20.40	0.73
	Sentinel-2	Simple Ratio	704 nm, 833 nm	0.13	24.87	0.60
	EnMAP		454 nm, 623 nm	0.11	19.74	0.74
	Sentinel-2	Reduced Simple Ratio	864 nm, 559 nm, 1610 nm	0.12	21.72	0.69
	EnMAP		1259 nm, 535 nm, 1609 nm	0.11	21.07	0.71
	Sentinel-2	NDI	704 nm, 833 nm	0.13	24.81	0.60
	EnMAP		482 nm, 599 nm	0.11	20.54	0.72
	EnMAP	Individual index	CIgreen	0.16	29.78	0.42
		Individual index	CIrededge	0.14	25.65	0.57
		Individual index	MCARI	0.13	23.75	0.63
		Individual index	MCARI1	0.21	39.43	−0.01
		Individual index	MCARI2	0.21	38.54	0.03
		Individual index	CAI	0.17	32.26	0.32
		Individual index	PSRI	0.16	30.02	0.42
	Sentinel-2	mSR	833 nm, 492 nm, 704 nm	0.11	20.42	0.73
		mND	704 nm, 833 nm, 492 nm	0.11	20.71	0.72
		3BSIV	704 nm, 739 nm, 492 nm	0.10	18.14	0.78
		3BSIW	833 nm, 492 nm, 704 nm	0.12	23.19	0.65
		3BSIT	704 nm, 492 nm, 833 nm	0.13	23.88	0.63

## Appendix E. Leave-one-out cross validation for the multivariate models selected with simulated annealing

**Table E1.** The median, relative median, standard deviation (sd), minimum (min), and maximum (max) root mean squared errors (RMSEs) and pseudo coefficients of determination (pseudo *R*^2^, squared correlation between observed and predicted) from 50 runs of leave-one-out cross validated (CV) simulated annealing variable selection per response variable (LAI, LAIeff, and CC) and number of predictors (2 and 3) derived from sentinel-2B MSI and EnMAP data.

		Sensor									
		Sentinel-2 MSI					EnMAP				
		CV RMSE					CV RMSE				
Response variable	Number of predictors	median	median [%]	sd	min	max	median	median [%]	sd	min	max
LAI	2	0.74	19.6	0.046	0.69	0.9	0.53	14.15	0.073	0.42	0.74
	3	0.73	19.22	0.548	0.6	4.52	0.42	11.15	0.123	0.30	1.17
LAIeff	2	0.58	22.23	7.181	0.53	51.36	0.38	14.58	0.043	0.31	0.48
	3	0.57	21.85	0.115	0.35	0.86	0.34	13.10	0.070	0.22	0.70
CC	2	0.08	15.35	0.005	0.08	0.1	0.08	15.82	0.007	0.07	0.10
	3	0.08	15.37	0.009	0.07	0.1	0.07	13.44	0.012	0.05	0.10
		CV pseudo *R*^2^					CV pseudo *R*^2^				
Response variable	Number of predictors	median		sd	min	max	median		sd	min	max
LAI	2	0.86		0.017	0.8	0.87	0.93		0.021	0.87	0.95
	3	0.86		0.102	0.19	0.91	0.95		0.023	0.82	0.98
LAIeff	2	0.84		0.125	0.13	0.86	0.93		0.015	0.90	0.95
	3	0.85		0.05	0.73	0.94	0.94		0.021	0.85	0.98
CC	2	0.85		0.018	0.78	0.87	0.84		0.028	0.77	0.89
	3	0.85		0.03	0.77	0.9	0.89		0.037	0.80	0.95
